# Electromagnetic Interference on Pacemakers

**Published:** 2002-07-01

**Authors:** Okan Erdogan

**Affiliations:** Department of Cardiology, School of Medicine, Trakya University, Edirne, Turkey

## Abstract

External sources, either within or outside the hospital environment, may interfere with the appropriate function of pacemakers which are being implanted all around the world in current medical practice. The patient and the physician who is responsible for follow-up of the pacing systems may be confronted with some specific problems regarding the various types of electromagnetic interference (EMI). To avoid these unwanted EMI effects one must be aware of this potential problem and need to take some precautions. The effects of EMI on pacemaker function and precautions to overcome some specific problems were discussed in this review article. There are many sources of EMI interacting with pacemakers. Magnetic resonance imaging creates real problem and should be avoided in pacemaker patients. Cellular phones might be responsible for EMI when they were held on the same side with the pacemaker. Otherwise they don't cause any specific type of interaction with pacemakers. Sale security systems are not a problem if one walks through it without lingering in or near it. Patients having unipolar pacemaker systems are prone to develop EMI because of pectoral muscle artifacts during vigorous active physical exercise.

Pacemakers (PM), either dual or single chamber, are currently being implanted in an increasing number for various indications all around the world. Although PM are very sophisticated and technically challenging devices, they may be affected by many internal and external factors. Patients with PM need to be regularly and carefully evaluated for any source of environmental factors that might interfere with pacemaker function. Patients who are dependent on pacemaker are especially at increased risk for developing adverse effects in case of an electromagnetic interference (EMI). EMI is generally defined as interference of pacemaker function by the signals generated by external sources. Current PM are relatively immune to EMI because the circuitry is shielded inside a hermetically sealed titanium or stainless steel case that often has an additional insulative coating. In addition, increased use of bipolar leads also decreased the susceptibility of pacing systems to EMI. Pacing systems are capable of filtering out some noncardiac signals by using bandpass filters that might prevent sensing of external signals responsible for EMI. Within the context of EMI which may be prevented by some precautions there are many external environmental sources to be discussed in this article.

## Common sources for EMI

### a)Transthoracic DC cardioversion

Among the problems caused by DC shock on pacemaker function some are to be mentioned: reversion to back-up mode, transient increases in capture threshold and loss of capture as well as destruction of the PM generator and circuitry [1-3]. A clinical study involving 36 patients with unipolar PM implanted on the right pectoral side revealed that 50 % of patients developed loss of capture due to increased stimulation thresholds. They received higher cumulative shock energies. Seven patients had sensing failure and three patients developed generator failure requiring replacement. The authors suggested that the utilized shock energy should be as low as possible and before shock attempt the PM should be programmed to its maximal output [2]. These problems can be prevented by placing the paddles or patches at least 15 cm away from the generator or using an anterior-posterior approach. The pacemaker should always be interrogated before and after the attempt of cardioversion.

### b) Radiofrequency catheter ablation

Radiofrequency (RF) generators produce unmodulated signals with frequencies between 400 and 500 kHz. Patients having Thera and Kappa model PM underwent RF ablation and exhibited excellent protection against interference produced by RF current. Pacing inhibition, under-or over sensing were not observed [4]. Pfeiffer et al [5] evaluated 25 patients with 13 different PM models, most of them with unipolar leads, during RF current delivery. In contrast to the previous study result they observed sensing failure in 8 and pacing failure in 4 patients. In general, patients with PM undergoing RF ablation have their PM checked before and after ablation. The dependency status should be ascertained and temporary pacing must be available. Rate response function should be turned off. RF applications should be as brief as possible and remote from the pacing electrode tip. If the patient is not dependent, the pacemaker can be programmed to OOO or VVI at a lower rate than the intrinsic heart rate. If the patient is dependent, the PM should be programmed to VOO mode and a temporary PM wire should be in place as back-up. Reinterrogation of the PM after the procedure is essential and integrity of the pacing circuit should be evaluated.

### c) Electrocautery

Electrocautery uses radiofrequency current to cut or coagulate tissues. It may produce signals that might inhibit the pacing stimuli or trigger ventricular pacing due to atrial oversensing. Additionally, current generated by the electrocautery can cause myocardial damage due to concentration of current at the electrode-tissue interface and subsequent elevation of pacing threshold. Use of the electrocautery tip close to the PM may cause it to revert to a noise reversion fixed rate mode or inhibit it due to oversensing of signals. Therefore, electrocautery should be bipolar, if possible and not be used in the vicinity of the PM. The orientation of current flow should be perpendicular to the lead of the PM system [6]. Electrocautery application should be limited to a few seconds. The PM should be programmed to asynchronous VOO mode and/ or a temporary PM should be inserted as back-up in case of dependency. Instead of electrocautery use of an ultrasonic scalpel reduces EMI [7] . Minimal power settings should be used for electrocautery. The heart rhythm should be monitored and the PM checked after the surgery.

### d) Magnetic resonance imaging

Magnetic resonance imaging (MRI) is a very important diagnostic tool and widely available today. MRI which is contraindicated in patients with PM utilizes strong electromagnetic fields that further interferes with PM function. Exposure to the magnetic field of MRI closes the reed switch which enables the PM function in the magnet mode and rate. Magnetic field may also cause significant torquing effect on the generator though reports about newer PM with little amount of ferromagnetic material in their construction revealed no significant physical movement [8,9]. A clinical prospective study conducted by Vahlhaus et al [10] comprised 32 patients with PM. Patients were exposed to MRI at 0.5 Tesla. Lead impedance, sensing and stimulation thresholds did not change after MRI. Battery voltage decreased immediately after MRI and recovered 3 months later. Battery current and impedance tended to increase. MRI affected neither PM programmed data, nor the ability to interrogate, program or telemetry. Temporary deactivation of the reed switch occurred in 12 patients in the center of the magnetic field. MRI at 0.5 Tesla did not cause irreversible changes in PM systems. Rapid pacing in unipolar systems exposed to the pulsing radiofrequency field owing to the antenna effect of the electrode system has been demonstrated [11]. Another potential adverse effect of MRI is electrode heating. MRI at 1.5 Tesla caused major temperature rise at the electrode-tissue interface [12]. In some patients who definitely need MRI study for any reason it might be cautiously undertaken with the following recommendations. The PM should be fully checked before and after MRI and dependency status should be evaluated. In non-PM dependent patient the device should be programmed to OOO, if it is available. Low magnetic field (0.5 Tesla) is preferable. The patient should be monitored by pulse oximetry, blood pressure, heart rate and electrocardiogram. My personal experience with non-pacemaker dependent patients undergoing head MRI confirmed no obvious signs of device failure and patient discomfort after the above mentioned precautions have been undertaken.

### e) Radiotherapy

High energy radiation may be responsible for many types of adverse effects such as direct damage of the PM circuitry or transient electromagnetic field effect. Modern PM use complementary metal oxide semiconductor circuitry which is very reliable, energy and space efficient. Radiation can cause damage to the thin oxide layers and transistors because of accumulation of positive charge inside the circuitry leading to failure of various battery components or accelerated battery depletion [13]. Type of radiation, cumulative dose and location of the device predicts the amount of damage. Changes in sensing capability, failure of telemetry function, runaway function and complete shut down may all occur [14-16]. Souliman et al [17] tested 18 PM during radiotherapy. They observed temporary change to safety mode pacing lasting for the duration of irradiation; change to interference mode pacing from which recovery may occur after reprogramming the PM; severe damage in which case the PM stops generating pulses. Recovery from this takes a long time. They recommended that patients undergoing radiotherapy be monitored closely during the course of the treatment and a few weeks thereafter. Some precautions need to be undertaken before exposing the patient with PM to radiotherapy, if it is absolutely necessary. The PM should be checked thoroughly and patients dependency status should be verified. One must position the field of radiation at an angle oblique to the PM in order to minimize the amount of radiation delivered at the PM site. A total accumulated dosage limit of 2 rad should be estimated using either luminescent dosimeters or a diode dose measurement system and additional shielding of the PM with a 1cm margin may be required. Direct irradiation of the PM must be avoided and if this is not possible, the PM should be explanted and moved to another suitable site [13]. Careful monitoring of the patient and temporary PM availability should be assured.

### f) Extracorporeal shock wave lithotripsy

Because most PM are implanted in the pectoral area, there is little chance of damage to the generator. If the shock wave is directly aimed at a PM, it can be damaged. The delivery of the shock wave should be timed to the ventricular stimulus output in patients who are being paced to avoid PM inhibition owing to oversensing of the shock wave [13]. The lithotriptor focal point should be placed at least 25 cm away from the PM which should be checked before and after the procedure.

### g) Cellular phones

Mobile or cellular phones may interact with PM function by inhibiting the pacing output, asynchronous pacing and ventricular triggering [18,19]. When the antenna is located near the pulse generator header, interaction may occur very easily. Hayes DL et al [20] published a landmark article which was a clinical, multicenter study comparing the effects and interaction of different cellular phones with PM. A total of 980 patients were included into the study. The incidence of all types of interference was 20%. Ventricular tracking of signals sensed on the atrial channel, noise reversion and inhibition of ventricular output were the most commonly observed types of interference. The incidence of overall clinical significant interference was 6.6 %. There was no clinically significant interference when the phone was placed in the normal position over the ear. Interference that was definitely clinically significant occurred in only 1.7 % of tests and only when the phone was held over the pacemaker. Interference was more common in dual chamber systems (25.3 %) than in single chamber systems (6.8 %) and in digital telephones (24%) compared to analog telephones (3 %). Patients who are PM dependent should use an analog type cellular phone system. Carrying the phone on the same side of the body as the implanted PM may cause interference. When using the phone, it should be held at least 15 cm away from the PM and on the opposite ear.

## Uncommon sources of EMI

Transcutaneous nerve stimulation is a method for pain relief used in medical facilities. In rare cases it may be sensed by the PM, especially in unipolar systems. However, a recent study showed that it can be safely used in PM patients [21]. Hospital pager systems may disturb telemetry in the form of inaccurate battery voltage, current and impedance measurements, disturbances in intracardiac electrogram tracings or total interruption of telemetric communications. The reason for EMI in this setting was an overlap of carrier frequencies of some PM programmers (32-37 kHz) with those of 36.11 kHz pager system [22]. Electromagnetic energy generated from a variety of dental instruments, including ultrasound scalers and cleaners, and electrosurgical instruments can also cause transient inhibition of pacemaker output [23]. Certain cardiac monitoring systems used for recording continuous electrograms in hospitalized patients can cause inappropriate rate changes in patients with rate adaptive pacing systems that use a minute ventilation sensor [24]. Antitheft devices are currently being used almost everywhere. They may cause EMI with pacemakers. A study revealed that acoustomagnetic systems are more responsible for EMI than the other systems [25]. Reversion to asynchronous pacing and rapid ventricular pacing owing to tracking of high frequency signals sensed in the atrial channel are the two most common observed interactions. They are transient and return to normal function once the patient was out of the field. Patients with any type of implanted electronic medical system should be forewarned: Dont lean, dont linger near any potential source of EMI [26]. Electric arc welding causes interference by the electrical fields generated by the welding electrode and magnetic fields generated by the large current flowing through the welding electrode or cable. In a recent study, patients working with arc welding demonstrated no interference of a specific PM model in certain conditions [27].

In conclusion, during our daily life patients with PM are potentially vulnerable to the adverse effects of many EMI sources. Physicians taking care about PM patients should be aware of these problems and precautions need to be undertaken for prevention of possible EMI.
